# Nutrients and phytochemicals characterisations, acute and sub-acute oral toxicity studies of BobyGuard C, a polyherbal nutraceutical with anti-breast cancer properties

**DOI:** 10.3389/ftox.2025.1598185

**Published:** 2025-05-01

**Authors:** Borelle Mafogang, Roger Ponka, Joseph Ngakou Mukam, Elie Fokou

**Affiliations:** ^1^ Department of Biochemistry, Faculty of Science, University of Yaoundé 1, Yaoundé, Cameroon; ^2^ Department of Agriculture, Livestock and Derived Products, The National Advanced School of Engineering, University of Maroua, Maroua, Cameroon; ^3^ Department of Animal Biology and Physiology, Faculty of Science, University of Yaoundé 1, Yaoundé, Cameroon

**Keywords:** BobyGuard C, standardization, nutrient, phytochemical, acute toxicity, subacute toxicity

## Abstract

**Background:**

In 2022, approximately 2.3 million new cases of female breast cancer and 670,000 related deaths worldwide despite significant advancements in conventional treatments. BobyGuard C (BGC) is a novel polyherbal nutraceutical formulated from five plants, selected for their antioxidant, anticancer, anti-inflammatory and nutritional properties to be used for breast cancer management. This study aimed to characterize its physicochemical, nutritional, and phytochemical properties as well as assess its safety through acute and sub-acute oral toxicity studies in Wistar rats.

**Methods:**

Thecomposition of BGC was analyzed for macronutrients, minerals, and phytochemicals using standard methods. Antioxidant activity was assessed through DPPH, TAC and FRAP assays, while antiproliferative activity was evaluated using the MTT assay on MDA-MB 231 and MCF-7 breast cancer cell lines. Acute (single 5,000 mg/kg dose with 14 days observation) and sub-acute oral (daily administration of 784, 1,568, and 3,136 mg/kg for 28 days) toxicity studies in female Wistar rats followed OECD guidelines.

**Results:**

BGC was found to be rich in proteins (38.36 g/100 g), carbohydrates (59.70 g/100 g), and essential minerals such as magnesium (60,066.67 µg/100 g), and it was free from toxic heavy metals. Several bioactive compounds, including diosgenin, diosbulbin H, β-carotene, Bafoudiosbulbin G and catechin were identified in BGC. Phytochemical analysis revealed high levels of phenols (9,783.48 mg GAE/100 g), flavonoids (47.72 mg QuE/100 g), and alkaloids (106.14 mg berberine eq/100 g), contributing to its strong antioxidant activity (DPPH inhibition: 90.39%). BGC exhibited significant antiproliferative effects on MDA-MB 231 cells, highlighting its potential anticancer activity. Acute toxicity tests showed no mortality at 5,000 mg/kg, with an LD_50_ exceeding this dose. In the sub-acute 28-day repeated-dose oral study, doses up to 3,136 mg/kg/day resulted in some dose dependent hematological and biochemical changes but no histopathological abnormalities were observed indicating its safety at lower doses.

**Conclusion:**

BGC is a nutritionally rich formulation with potent antioxidant and anticancer potential, demonstrating a favorable safety profile at lower dose (784 mg/kg).

## 1 Introduction

In 2022, there were approximately 2.3 million new cases of female breast cancer and 670,000 related deaths worldwide. By 2050, the number of new cases is projected to rise by 38%, and deaths by 68%, with the greatest burden expected to fall on low-HDI countries (Kim et al., 2025). Despite advances in conventional treatments, the fight against breast cancer continues to face challenges such as high treatment costs, adverse side effects, and drug resistance (Burguin et al., 2021; Barrios, 2022). These challenges have fueled the search for alternative, more affordable, and safer therapeutic options, including nutraceuticals, which are food-derived products with health benefits beyond basic nutrition. Nutraceuticals, especially polyherbal formulations, are increasingly being explored for their potential role in cancer prevention and therapy (Fayez et al., 2022). Polyherbal formulations, which combine multiple herbs with medicinal properties, have been used in traditional medicine systems for centuries. These formulations are believed to provide synergistic therapeutic effects, where the combined bioactive compounds from different plants act on multiple molecular targets, offering a more comprehensive approach to disease management. In the context of cancer, polyherbal nutraceuticals hold significant promise due to their potential antioxidant, anti-inflammatory, and anticancer and anticancer enhancing properties (Maiuolo et al., 2021). HC9, a polyherbal formulation, retards tumor growth in mouse melanoma model and induces immunomodulation (Kaul-Ghanekar et al., 2020). Also, Jaeumkanghwa-tang has been demonstrated to reduce the risk of developing tamoxifen resistance, risk of recurrence and protects the uterus against the adverse effects of tamoxifen (De Oliveira Andrade et al., 2019). However, while polyherbals offer great potential, one of the major challenges in their development and use is ensuring consistent quality, safety, and efficacy.

BobyGuard C is a novel polyherbal nutraceutical developed to upgrade the anticancer properties of multiple foods traditionally used in herbal medicine. Preclinical studies have shown that BobyGuard C possesses various biological activities, including antioxidant, antiproliferative, and anti-inflammatory effects, which may contribute to its potential role in breast cancer management (Mafogang et al., 2024). The formulation includes a combination of herbs such as *Adansonia digitata, Brassica oleracea var f alaba, Afrostyrax lepidophyllus, Spirulina platensis and Dioscorea bulbifera* with rich profile of bioactive compounds, such as flavonoids, terpenoids, and alkaloids, known for their potential to inhibit cancer cell growth, induce apoptosis, and modulate key signaling pathways involved in cancer progression (Moukette et al., 2015; Eltahir and Elsayed, 2019; Mandrich and Caputo, 2020; Wang et al., 2023; Spínola et al., 2024). These herbs have also shown protective effects in cancer models. Spirulina reduced breast tumor incidence in rats from 87% to 13% (Xin et al., 2023), while baobab fruit pulp extracts induced cancer cell apoptosis (Elsaid, 2013). *Dioscorea bulbifera* extracts exhibited anti-inflammatory and analgesic effects by inhibiting mediators like histamine and prostaglandins (Mbiantcha et al., 2010). White cabbage extract protected against liver injury via antioxidant action (Morales-López et al., 2016), and *A. lepidophyllus* bark mitigated doxorubicin-induced cardiotoxicity in cell models (Moukette et al., 2021). However, before its potential clinical application, a thorough characterization and safety evaluation of BobyGuard C are essential.

Characterizing polyherbal formulations like BobyGuard C is essential to ensure consistency, quality, and safety. The therapeutic effects depend on both individual bioactive compounds and their interactions, which can vary due to factors like herb source and extraction methods (Mosihuzzaman and Choudhary, 2008). Rigorous toxicity testing, including acute and sub-acute studies, is critical to determine safe dosage ranges and identify potential adverse effects (Mosihuzzaman and Choudhary, 2008). The present study aims to comprehensively characterize BobyGuard C, assessing its physicochemical and phytochemical properties to establish its composition. Additionally, the study evaluates the acute and sub-acute oral toxicity of BobyGuard C to assess its safety profile in animal models.

## 2 Materials and methods

### 2.1 Materials and reagents

Quercetin dihydrate, 1,1-diphenyl-2-picrylhydrazyl (DPPH) radical, gallic acid, butylated hydroxytoluene (BHT), Folin-Ciocalteu phenol reagent, L-ascorbic acid, 2,4,6-tris(2-pyridyl)-s-triazine (TPTZ), aluminum chloride (AlCl_3_), sodium carbonate (Na_2_CO_3_), sodium hydroxide (NaOH), potassium persulfate, iron (II) sulfate, glacial acetic acid, hydrochloric acid (HCl), sulfuric acid (H_2_SO_4_), and ammonium molybdate were used. Fetal bovine serum (FBS), the 3-(4,5-dimethylthiazol-2-yl)-2,5-diphenyltetrazolium bromide (MTT) dye reduction assay kit, streptomycin and penicillin, non-toxic GLPBio Cell Counting Kit-8 (CCK-8) were procured. The Annexin V-FITC Apoptosis detection and cycle TEST PLUS DNA reagent kits were also used. All other chemicals and reagents were of analytical grade.

The ingredients of BobyGuard C were obtained from various locations in Cameroon and identified at the Cameroon National Herbarium (CNH) by Mr. Ngansop, a botanist, using reference materials (Table 1).

**TABLE 1:** Entrants’ characteristics and BobyGuard C composition.

Common name/local name	Botanical name	Identification number	Part used	Zone of collection	Extraction yield (%)	Proportion by weight in BGC (%,w/w)
Baobab	*Adansonia digitata*	7815 SRF/Cam	Fruit pulp	Benoue, North region 9^°^10′38.52″N 13°26′18.24″E	NA	20
White cabbage	*Brassica oleracea* var. *capitata f. alba*	25,686 SRF/Cam	Leaves	Menoua, West region 5°27′59.99″N 10°06′60.00″E	2.20	9
Country/bush onion	*Afrostyrax lepidophillus*	39,020 SRF/Cam	Bark	2°04′31.51″N 15°17′1.63″E	1.52	6
Air potato	*Dioscorea bulbifera*	29,684 SRF/Cam	Bulbils	Bamendjou, West region 5°23′21.85″N 10°19′52.34″E	13.61	26
Spirulina	*Spirulina platensis*	NA	Whole algae	Nomayos, Centre region 3°47′24.39″N 11°26′25.55″E	NA	38

NA: not applicable.

### 2.2 Preparation of BobyGuard C

#### 2.2.1 Preparation of ingredients

##### 2.2.1.1 Spirulina (*Spirulina platensis)*

Fresh spirulina was dried in an oven at 40°C for 48 h following the procedure of Moor et al. (2016).

##### 2.2.1.2 Baobab (*Adansonia digitata*)

The baobab fruit was processed according to Maptouom et al. (2020), with modifications. The fruit was dehusked to obtain the seeds and pulp. The pulp was separated from the seeds by pounding it in a traditional mortar. The resulting pulp was sieved (sieve No. 40) to remove fibers, and the pulp powder was stored in dark plastic bags.

##### 2.2.1.3 White cabbage (*Brassica oleracea* var. *capitata f. alba*)

Fresh white cabbage extract was prepared following Tanongkankit et al. (2013). The cabbage leaves were chopped and mixed with deionized water at a ratio of 1:1.5 (w/v). The mixture was blended and microwaved at 390 W for 3 min. It was then filtered through Whatman No. 4 paper, and the filtrate was dried in a ventilated oven at 45°C for 72 h to obtain dry cabbage extract.

##### 2.2.1.4 Air potato *(Dioscorea bulbifera)*

The air potato bulbils were processed based on Wang et al. (2009), with modifications. The bulbils were cleaned, chopped, and dried at 45°C for 48 h. The dried bulbils were then ground into powder and soaked in 70% ethanol (2:5 w/v) for 72 h at room temperature with occasional stirring. The mixture was filtered using Whatman No. 4 paper, and the filtrate was evaporated and oven-dried at 45°C until fully dry.

##### 2.2.1.5 Country/bush onion (*Afrostyrax lepidophyllus)*

The fresh bark of country/bush onion was collected and processed following Moukette et al. (2015), with adjustments. The bark was shade-dried at room temperature and ground into powder. The powder was macerated in a water/ethanol mixture (30:70 v/v) at a 1:5 (w/v) ratio for 48 h. The extract was filtered through Whatman No. 4 paper, and the residue was re-extracted using the same method. The combined filtrates were evaporated and oven-dried at 45°C for 48 h.

Baobab pulp powder and all dried extracts were stored separately in conical tubes at −20°C, while spirulina was kept in an airtight container at room temperature.

The extraction yield was calculated using the following formula (Equation 1)

$$\text{Extraction yield}=\left(\text{Dry extract weight}\right)/\left(\text{Raw material weight}\right)\times 100$$ (1)

#### 2.2.2 Development of BobyGuard C

BGC was prepared by combining the different ingredients in specific proportions based on the minimum effective doses reported in the literature for each ingredient’s desired effect (as detailed in Table 1). The ingredients were thoroughly mixed and blended, followed by drying the blend at 45°C for 24 h. After drying, the mixtures were blended again and stored in airtight containers at room temperature.

### 2.3 Physico-chemical characterization of BobyGuard C

The physico-chemical evaluation of BGC involved assessing characteristics such as percentage loss on drying, solubility, total ash, pH, and extractive values (Shaikh et al., 2016; Mastan, 2020).

#### 2.3.1 Percentage of loss on drying

A precise weight of 3 g of BGC (M1) was measured in an evaporating dish. The sample was dried at 105°C for 5 h and then reweighed (M2). The percentage loss on drying was calculated using the following formula (Equation 2)

$$\text{Percentage loss on drying}=\left(\left(M1–M2\right)/M1\right)\times 100$$ (2)

#### 2.3.2 Total ash determination

BGC was placed in a silica dish and incinerated in a furnace at 550°C until the sample turned white. The total ash content was calculated using the formula (Equation 3)

$$\text{Total ash}=\left(\left(\text{Weight of ash}\right)\;/\;\left(\text{Initial weight of nutaceutical}\right)\right)\times 100$$ (3)

#### 2.3.3 pH determination

Solutions of BGC (1% and 10%) were prepared in 2% ethanol and allowed to stand for 30 min before measuring the pH.

#### 2.3.4 Solubility test

In a dry test tube, 1 g of BGC was weighed, and 5 mL of solvent (ethanol, water, or ethyl acetate) was added. The mixture was thoroughly shaken for 30 min using a magnetic stirrer, and the solutions were observed for solubility in different solvents.

#### 2.3.5 Determination of alcohol- and water-soluble extractives

Approximately 3 g of BGC was mixed with 50 mL of ethanol (to assess alcohol-soluble extractives) in a closed flask and stirred for 5 h using a magnetic stirrer. The extract was then filtered without solvent loss and dried at 105°C until a constant weight was achieved. This procedure was repeated using water to evaluate the water-soluble extractive. The extractive content was calculated using the formula (Equation 4)

$$\text{Alcohol }|\text{ water soluble extractive}=\left(\left(\text{Dry extract weight}\right)/\left(\text{Sample weight}\right)\right)\times 100$$ (4)

### 2.4 Macronutrient, crude fiber, and vitamin C contents of BobyGuard C

#### 2.4.1 Macronutrient and crude fiber contents

The macronutrient (protein, lipid, carbohydrate) and crude fiber contents of BobyGuard C (BGC) were analyzed using AOAC standard methods (ACS, 1992), with results expressed as percentages (g/100 g).

##### 2.4.1.1 Lipid content

Lipid content was determined using the Soxhlet method. A 2 g sample was placed on pre-weighed filter paper, sealed, and extracted with hexane in a Soxhlet apparatus at 70°C for 8 h. After drying at 105°C for 3 h, the sample was cooled, weighed, and the lipid percentage was calculated. Each sample was analyzed in three trials.

##### 2.4.1.2 Protein content

Crude protein content was assessed using the Kjeldahl method. About 2 g of the sample was digested with a mixture of CuSO_4_, K_2_SO_4_, and sulfuric acid at 420°C until colorless. After dilution, the sample underwent steam distillation, followed by titration with sulfuric acid to determine nitrogen content, which was then converted to protein using a factor of 6.25. This procedure was repeated three times to ensure accuracy.

##### 2.4.1.3 Carbohydrate content

Total sugar content was measured using a glucose standard curve. A 1-g dried sample was boiled with 20 mL of distilled water for an hour, filtered, and treated with zinc sulfate and potassium ferricyanide. The resulting solution was adjusted to volume in a volumetric flask. The mixture was then reacted with phenol and concentrated H_2_SO_4_, developing a stable yellow color. Absorbance was read at 490 nm, with three trials conducted per sample.

##### 2.4.1.4 Crude fiber content

Crude fiber was measured by treating 0.5 g of defatted dry food with 30 mL of H_2_SO_4_ (0.26 N) and heating for 30 min. After cooling and filtering, KOH (0.23 N) was added and heated again. The residue was dried, calcined at 550°C, and weighed before and after incineration. Each sample was tested three times. Crude fiber content was calculated using Equation 5 below

$$\text{Crude fiber content}=\left(P2-P3\right)/P1\times 100$$ (5)

##### 2.4.1.5 Energy content

Energy content was calculated based on the caloric values of macronutrients: 4 kcal per Gram of carbohydrates and protein, and 9 kcal per Gram of fat. The formula (Equation 6) used was:

$$\text{Energy }\left(\text{kcal}\right)=\left(\text{Total carbohydrate }\left(g\right)\times 4\text{ kcal}\right)+\left(\text{Total protein }\left(g\right)\times 4\text{ kcal}\right)+\left(\text{Total lipid }\left(g\right)\times 9\text{ kcal}\right)$$ (6)

#### 2.4.2 Vitamin C content of BobyGuard C

The vitamin C content was determined using a titrimetric method (ACS, 1992). A 0.5 g sample of polyherbal nutraceutical powder was mixed with 10 mL of 90% acetic acid and stirred for 30 min. The solution was filtered into a 20 mL flask and topped off with 90% acetic acid. A 5 mL portion of the 10-fold diluted filtrate was mixed with 1 mL of glacial acetic acid in a test tube and titrated with 2,6-dichlorophenolindophenol (DCPIP) until a faint, stable pink color appeared. The volume of the titrant (T) was recorded. The process was repeated using 5 mL of water for the blank (B_1_) and 5 mL of ascorbic acid solution as a standard. Three trials were performed for each sample. The vitamin C content was calculated using Equation 7

$$\text{Vitamin }C=\left(\left(\text{Sample}-\text{Blank}\right)\;/\;\left(\text{Standard}-\text{Blank}\right)\right)\times \text{dilution factor}$$ (7)

### 2.5 Heavy metals and mineral content of BobyGuard C

Heavy metals (lead, cadmium, mercury and arsenic) and minerals (magnesium, manganese, zinc, copper, potassium, chlorine, iron, phosphorus and sulphur) in BGC were analyzed using energy dispersive X-ray fluorescence (EDXRF) (Tezotto et al., 2013). A 2 g sample of BGC was placed in a PANalytical Epsilon 3XLe apparatus, exposed to excitation for 300 s, and analyzed using a spectrometer equipped with a solid Si-pin-diode X-ray detector with a 140 µm Be window. Quantitative analysis was done via Omnian-standardless software.

### 2.6 Phytochemical screening

Phytochemical screening was conducted to identify classes of secondary metabolites present in BGC. An aqueous methanolic extract (20:80 v/v) was prepared by mixing 2 g of BGC with 25 mL of solvent and stirring for 3 h with a magnetic stirrer. The solution was then filtered through Whatman paper No. 4, and the filtrate was used for the following analyses (Shaikh and Patil, 2020):

#### 2.6.1 Alkaloids (Mayer’s test)

A solution of 60 mL mercuric (II) chloride (2.27% w/v) and 10 mL potassium iodide (50% w/v) was prepared and diluted to 100 mL. The presence of alkaloids was indicated by the formation of a white or yellowish precipitate.

#### 2.6.2 Saponins (foam test)

A 0.5 g sample was mixed with 10 mL of distilled water and agitated. Persistent foam formation after heating in a water bath for 5 min indicated the presence of saponins.

#### 2.6.3 Tannins (ferric chloride test)

A 6 mL solution was prepared in hot distilled water (0.17% w/v) and filtered. The filtrate was divided into three portions, which were mixed with 0.9% sodium chloride (NaCl), a mixture of NaCl and 1% gelatin, and ferric chloride. The presence of tannins was confirmed by a precipitate in the second portion and a blue, blue-black, or greenish color.

#### 2.6.4 Glycosides (Bontrager’s test)

A 1 mL sample solution was mixed with 1 mL of 5% sulfuric acid and heated in a water bath. The filtrate was then mixed with chloroform and left to stand for 5 min. Afterward, the lower chloroform layer was mixed with dilute ammonia. The appearance of a rose pink to red color indicated the presence of anthraquinone glycosides.

#### 2.6.5 Flavonoids (ammonia test)

A 5 mL diluted ammonia solution and concentrated sulfuric acid were added to 1 mL of the sample solution. The appearance of a yellow color confirmed the presence of flavonoids.

#### 2.6.6 Steroids (Salkowski’s test)

Concentrated sulfuric acid was added to 5 mL of the sample solution. The presence of steroids was indicated by a red color in the lower layer.

#### 2.6.7 Triterpenoids (Salkowski’s test)

Concentrated sulfuric acid was added to 5 mL of the sample solution. A golden yellow layer at the bottom indicated the presence of triterpenoids.

#### 2.6.8 Phenols (lead acetate test)

A 5 mL sample solution was mixed with 3 mL of 10% lead acetate. The formation of a white precipitate confirmed the presence of phenols.

#### 2.6.9 Anthocyanins (Borntrager’s test)

A 2 mL sample solution was mixed with 2 mL of 2N HCl and 2 mL of ammonia. The presence of anthocyanins was confirmed by a pink solution turning blue-violet upon the addition of ammonia.

#### 2.6.10 Reducing sugar (Fehling’s test)

A 1 mL sample of Fehling’s solution A and B was added to 1 mL of the sample solution and boiled in a water bath. A red precipitate indicated the presence of reducing sugars.

### 2.7 Phytochemical quantification and antioxidant/antiradical activities of BobyGuard C

#### 2.7.1 Test with ethanolic extracts

##### 2.7.1.1 Ethanolic extract preparation

BGC was extracted with 70% ethanol (1:10 w/v), stirred for 2 h, and filtered through Whatman No. 4 paper. The resulting filtrate was used for analysis.

##### 2.7.1.2 Total phenol content

Phenol content was measured using the Folin-Ciocalteu method (Singleton and Rossi, 1965). A 0.5 mL sample was mixed with 1 mL of 10% 2N Folin reagent, incubated for 5 min, then 1 mL of 10% Na_2_CO_3_ was added. After a 15-min incubation, absorbance was recorded at 765 nm. Results were expressed as grams of gallic acid equivalent per 100 g of dry weight (g GAE/100 g).

##### 2.7.1.3 Alkaloid content

A 1 mL extract (1 mg/mL) was mixed with 0.1 mL FeCl_3_ (2.5 mM in 0.5 M HCl) and 0.1 mL 1,10-phenanthroline. The mixture was incubated at 70°C for 30 min and absorbance was measured at 500 nm. Alkaloid content was expressed as mg berberine equivalents per Gram of dry extract (mg berberine eq/g) (Singh et al., 2004).

##### 2.7.1.4 Flavonoid content

A 0.1 mL extract was combined with 0.3 mL of distilled water and 0.03 mL of 5% NaNO_2_. After 5 min, 30 µL of 10% AlCl_3_ was added, followed by 0.2 mL of 1 mM NaOH after another 5 min. Absorbance was measured at 510 nm, and flavonoid content was calculated using a quercetin calibration curve, expressed as mg quercetin equivalents per Gram of dry extract (mg quercetin eq/g) (Kumaran and Karunakaran, 2005).

##### 2.7.1.5 Flavonol content

A 2 mL sample (0.1 mg/mL) was combined with 2 mL of 2% AlCl_3_ and 3 mL of sodium acetate solution (50 g/L), incubated for 2 h and 30 min at 20°C, and measured at 430 nm. Flavonol content was expressed as mg quercetin equivalents per Gram of dry extract (mg quercetin eq/g) (Kumaran and Karunakaran, 2005).

##### 2.7.1.6 DPPH assay

BGC’s antioxidant activity was assessed using the DPPH radical method (Kumaran and Karunakaran, 2005). A 50 µL sample was mixed with 2 mL of DPPH solution (0.05 mM) and incubated for 30 min in the dark. Absorbance was measured at 517 nm, and the inhibition percentage was calculated using Equation 8

$$\%\text{ Inhibition}=\left(\left(\text{Ab}0-\text{Ab}1\right)\;/\text{ Ab}0\right)\times 100$$ (8)

where Ab0 is the control’s absorbance and Ab1 is the sample’s absorbance.

##### 2.7.1.7 FRAP assay

This assay measures the reduction of ferric ions (Fe^3+^) to ferrous ions (Fe^2+^). A 0.1 mL extract was mixed with 3 mL of FRAP reagent, incubated for 5 min, and absorbance was read at 593 nm. Results were expressed as mg iron sulfate equivalent per 100 g of sample (Karthivashan et al., 2013).

##### 2.7.1.8 Hydroxyl radical scavenging activity

This activity was measured using the Fenton reaction (Wenli et al., 2003). A mixture containing 1.5 µL of extract, FeCl_3_, phenanthroline, phosphate buffer, and H_2_O_2_ was incubated, and absorbance was measured at 560 nm. The inhibition percentage was calculated using Equation 9

$$\%\text{ Inhibition}=\left(\left(\text{Ab}0-\text{Ab}1\right)\;/\text{ Ab}0\right)\times 100$$ (9)

##### 2.7.1.9 Total antioxidant capacity (TAC)

TAC was assessed using the phosphomolybdenum method (Prieto et al., 1999). A 0.3 mL sample was mixed with 3 mL of phosphomolybdate reagent, incubated at 95°C for 90 min, and absorbance was measured at 695 nm. TAC was expressed as mg ascorbic acid equivalents per Gram of extract (mg AAE/g extract).

#### 2.7.2 Test with other extracts

##### 2.7.2.1 Carotenoids

Carotenoid levels were determined using the method by Russell et al. (1935). Approximately 1 g of the sample was triturated with petroleum ether and transferred to a 20 mL flask, then filled to the mark with petroleum ether. Absorbance was measured at 450 nm using a spectrophotometer, with a control vial containing only petroleum ether. The carotene content (expressed as β-carotene) in mg/100 g of the product (Qc) was calculated with the formula (Equation 10)

$$\text{Qc}=\left(\varepsilon \;/\;2650\right)\times 10^{5}$$ (10)

where ε is the specific extinction coefficient of the solution in g/100 mL at its maximum absorption, calculated as ε = OD/C × l, where OD is the optical density, l is the cell thickness, and C is the concentration of the product in g/100 mL of petroleum ether.

##### 2.7.2.2 Phytate

Phytate content in BGC was measured according to the method by Unuofin et al. (2017). A 0.5 g sample was mixed with 100 mL of 2% HCl and shaken for 3 h. The mixture was filtered, and 25 mL of the filtrate was placed in a 250 mL flask. To this, 5 mL of 0.3% ammonium thiocyanate solution (as an indicator) and 53.5 mL of distilled water were added to adjust the acidity. The solution was titrated with iron III chloride (0.00195 g Fe per mL) until a persistent brownish-yellow color appeared. Phytate content was calculated using the formula (Equation 11)

Phytate content=Titrate×0.00195×1.19×100 (11)

##### 2.7.2.3 Tannins

For tannin content, a 0.125 g sample was extracted with 2.5 mL of 95% ethanol, stirred for 30 min, and filtered. The extract was centrifuged at 3,000 rpm for 10 min, and the supernatant was used for the analysis. A 0.5 mL portion of the extract was combined with 0.1 mL of ferric reagent (ferric ammonium sulfate in 2N HCl) and 3 mL of butanol-HCl reagent. The mixture was incubated at 100°C for 1 h, and absorbance (Ab) was read at 550 nm. Condensed tannins were expressed as leucocyanidin equivalents, using the formula (Equation 12) (Porter et al., 1985):

$$\text{Tannin content}=\left(\text{Ab}\times 78.26\times \text{Dilution factor}\right)\;/\;\left(\%\text{ Dry sample weight}\right)$$ (12)

##### 2.7.2.4 Oxalates

A 0.5 g sample was placed in an Erlenmeyer flask with 75 mL of 3M H_2_SO_4_ and stirred for 1 h. The mixture was filtered, and 25 mL of the filtrate was heated at 80°C–90°C for 1 h, then maintained at 70°C. The hot sample was titrated with 0.05 M KMnO_4_ until a pale pink color persisted. The oxalate content was calculated using the formula (Equation 13) (Unuofin et al., 2017):

$$\text{Oxalate content}=\left({\text{KMnO}}_{4}\;\left(0.05\;M\right)\times 2.2\right)\;/\;\left(\text{Dry sample mass}\right)\times 100$$ (13)

### 2.8 LC-MS analysis

Liquid chromatography-mass spectrometry (LC-MS) was performed using the Ultimate 3,000 system (Thermo Scientific) equipped with a standard autosampler. Separation was carried out on an Accucore C18 Reversed Phase UHPLC column (50 × 2.1 mm, 2.6 µm, 150 Å) at 35°C, with a flow rate of 0.4 mL/min. The mobile phases consisted of water with 0.1% formic acid (A) and acetonitrile with 0.1% formic acid (B). The injection volume was 5 μL, and a multistep linear gradient was used: 5% B isocratic for 5 min, 5% B to 60% B for 7 min, 60% B isocratic for 5 min, 60% B to 95% B for 4 min, followed by 95% B isocratic for 2 min. The initial conditions were maintained for 1 min. Detection was performed using a Diode Array Detector (DAD) at 190–600 nm. The system was coupled to a Q-TOF mass spectrometer (Bruker Daltonics) operating in positive ion mode with a capillary voltage of +4.5 kV. The MS/MS collision energy was set at 40 eV, and nitrogen was used as the drying, nebulizing, and collision gas. Data analysis was conducted using Data Analysis 4.3 software (Bruker Daltonics), which generated molecular formula predictions and compounds were identified by tentative method based on mass spectra and library matching.

### 2.9 *In vitro* cytotoxicity of BobyGuard C

#### 2.9.1 Cell lines and cell cultures

Human estrogen-sensitive (MCF-7) and non-sensitive (MDA-MB 231) breast cancer cells were cultured in RPMI-1640 medium supplemented with 10% FBS and 1% penicillin (100 U/mL)/streptomycin (100 g/mL). Incubation was carried out in a humid 5% CO_2_ incubator maintained at 37°C and 7.4 pH. Cell passage involved replacing 90% of the supernatant with fresh medium every 2 days. Before each experiment, the number of viable cells was estimated using an ELISA Multiskan TECAN reader counter system.

#### 2.9.2 Assessment of cell growth

Cell growth was assessed by the MTT dye reduction protocol. MCF-7 and MDA-MB 231 cells (100 μL, 1 × 10^5^ cells/mL) were seeded onto 96-well plates. The extract dissolved in DMSO was evaluated at concentrations of 12.5–200 μg/mL. The control was treated with the vehicle (DMSO 0.01%). At time points 0, 24, 48 and 72 h, 10 μL of MTT solution (5 mg/mL) was introduced and the cells were then incubated at 37°C, 5% CO_2_ for 2 h. Following that, the cells underwent lysis in a buffer composed of 10% SDS in 0.01 M HCl for an additional 2 h. The absorbance at 550 nm for each well was measured using a microplate ELISA TECAN^©^ SPARK reader. The cell growth percentage was computed as follows (Equation 14)

$$\%\;Cell\;growth=\left(\left(Ab550\;of\;treated\;cells\right)/\left(Ab550\;of\;control\;cells\right)\right)\times 100$$ (14)

#### 2.9.3 Assessment of cell proliferation

The inhibitory effect of BGA and BGB on cell proliferation was determined using the stable and CCK-8. MCF-7 and MDA-MB 231 cells (100 μL, 1 × 10^4^ cells/mL) were plated on 96-well plates and incubated at 37°C with 5% CO_2_ for 24 h. Afterward, 10 µL of the sample was tested at concentrations ranging from 50 to 100 μg/mL, while the control was treated with the vehicle (DMSO 0.01%). Following 48 h of incubation, 10 µL of CCK8 solution was introduced into each well of the plate and allowed to incubate for 4 h (5% CO_2_, 37°C). The plates were gently homogenized on a shaker before reading the absorbance at 450 nm wavelength utilizing a microplate TECAN reader.

### 2.10 Acute and subacute toxicity evaluation

#### 2.10.1 Animals and husbandry

Female nulliparous Wistar rats, aged between 50 and 56 days, were obtained from the Animal Physiology Laboratory at the University of Yaoundé I, Cameroon. The rats were housed in plastic cages (five animals per group) at room temperature and had free access to standard rat chow and water *ad libitum*. The animals were maintained under standard environmental conditions (23°C–25°C, 12 h/12 h light/dark cycle). Animals were acclimatized to laboratory environment for a week prior to start study. Their diet consisted of corn (36.7%), fish flour (4.8%), bone flour (14.5%), wheat (36.6%), crushed palm kernel (7.3%), a vitamin complex (Olivitazol®- 0.01%), and sodium chloride (0.3%).

All procedures involving the animals adhered to ethical guidelines set by the Institutional Ethics Committee of the Ministry of Scientific Research and Innovation of Cameroon, in line with European Union regulations on animal care (EEC Council 86/609). Ethical approval (BTC-JIRB2022-025) was granted by the Joint Institutional Review Board for Animal and Human Bioethics at the University of Yaoundé I.

#### 2.10.2 Dose calculation

Doses for the toxicity study were selected based on the effective dose of a polyherbal formulation used in breast cancer treatment (Mafogang et al., 2024). For the sub-acute toxicity study, higher doses (784, 1,568 and 3,136 mg/kg/day) were administered. Doses were calculated according to the individual body weights of the rats prior to the study.

#### 2.10.3 Acute toxicity study

The acute oral toxicity of BGC was assessed in female rats following OECD Guideline No. 423 (OECD, 2009). BGC was dissolved in 2% ethanol. After an overnight fast, the rats received a single oral dose of 5,000 mg/kg body weight (BW) of BGC. Observations for clinical signs or mortality were made frequently during the first 24 h (0.5, 1, 2, 3, 4, and 24 h). Since no mortality or signs of toxicity were observed, the procedure was repeated on another group of rats. Biweekly weight measurements and daily observations focused on mortality, toxicity, behavioral changes, and physical signs (skin, eyes, fur, mucous membranes, respiration, and motor activity) for 14 days. Additional symptoms such as sleep disturbances, diarrhea, tremors, and coma were monitored. At the end of the study, all rats were sacrificed for necropsy.

#### 2.10.4 Subacute toxicity study

The subacute oral toxicity of BGC was examined in female rats according to OECD Guideline No. 407 (OECD, 2008). The rats were divided into four groups (five rats per group) and were administered either 2% ethanol (control) or BGC at doses of 784, 1,568 and 3,136 mg/kg BW for 28 days. Body weight was recorded biweekly, and daily observations focused on mortality, toxicity, behavioral changes, and physical signs (skin, eyes, fur, mucous membranes, respiration, and motor activity). Additional symptoms like sleep disturbances, diarrhea, tremors, and coma were monitored. After 28 days, surviving animals were decapitated following a 12-h fast under anesthesia (ketamine and diazepam), and blood samples were collected for hematology and biochemistry analyses. The heart, liver, lungs, spleen, kidneys, and reproductive organs were weighed, and relative organ weights were calculated. Selected organs (liver, kidneys, heart, spleen, ovaries, uterus, brain and lungs) were preserved in 10% formalin for histological analysis.

##### 2.10.4.1 Hematological analysis

Blood morphology was analyzed using the MINDRAY BC-2800 Auto Hematology Analyzer (Shenzhen Mindray Bio-medical Electronics^®^, Shenzhen, China). Parameters evaluated included WBC count, RBC count, PLT count, Hb, HCT, MCV, MCH, and MCHC. Deviation from normal values (%) was also calculated. The degrees of variation (decrease or increase) were estimated for three severity degrees (slight = grade 1, moderate = grade 2, severe = grade 3) as an increase or decrease of 10% (slight), 50% (moderate), and 70% (severe) referring to the respective acceptable ranges. For these parameters, the division of severity into 10%, 50%, and 70% was chosen because they can be correlated with pathological findings, for example, a deviation of 10% in HCT or Hb usually has no effect in the histopathological examination. But a deviation of more than 10% becomes visible as either extramedullary hemopoiesis or bone marrow atrophy, or embolism. A deviation of 70% and more is usually associated with severe damage or even death (De Kort et al., 2020).

##### 2.10.4.2 Biochemical parameter analysis

Biochemical analyses were performed via spectrophotometry. Protein levels in mammary gland and tumor homogenates were measured using the Gornall method (Gornall et al., 1949). Serum levels of ALT, ALP, AST, creatinine, albumin, cholesterol, and triglycerides were determined following the manufacturer’s instructions (LABKIT).

##### 2.10.4.3 Histological analysis

Tissue samples from the liver, kidney, heart, spleen, ovaries, uterus, brain and lung were dehydrated, embedded in paraffin, sectioned into 4–5 μm slices, and stained with hematoxylin and eosin for microscopic examination. Histological assessments were conducted using an Axioskop 40 microscope and a Model N-400ME photomicroscope, with tissues observed at ×40 and ×100 magnifications.

### 2.11 Statistical analysis

Results were presented as mean ± standard error of the mean (SEM). Comparisons between groups were conducted using one-way ANOVA followed by Dunnett’s *post hoc* test, with p < 0.05 considered statistically significant. Analyses were performed using GraphPad Prism 8 and R Studio version 4.21.

## 3 Results

### 3.1 Physicochemical properties of BobyGuard C

Table 2 outlines the physicochemical properties of BobyGuard C, such as loss on drying (11.67%), ash content (6.87 g/100 g), and water/alcohol-soluble extractives (34.39% and 29.61%, respectively). It also presents pH values of 6.25 ± 00 and 5.72 ± 02 at 1% and 10% concentrations, respectively, both of which indicate slight acidity. Furthermore BobyGuard C is soluble in water, ethanol, and ethyl acetate, making it versatile in different solvents.

**TABLE 2:** Physicochemical characteristics of BobyGuard C.

Formulation	Loss on drying (%)	Ash (g/100 g)	Water soluble extractive (%)	Alcohol soluble extractive (%)	pH (1%)	pH (10%)	Solubility in water	Solubility in ethanol	Solubility in ethyl acetate
BGC	11.67 ± 0.01	6.87 ± 0.06	34.39 ± 0.20	29.61 ± 0.64	6.25 ± 00	5.72 ± 02	Soluble	Soluble	Soluble

Values are expressed as mean ± SEM.

### 3.2 Nutrient content of BobyGuard C

BGC is protein-rich (38.36 g/100 g), with carbohydrates (59.70 g/100 g) being the dominant macronutrient. It contains small amounts of lipids (3.16 g/100 g), crude fibre (2.46 g/100 g), and energy (420.70 kcal/100 g), indicating that BobyGuard C is nutritionally dense. Vitamin C content is relatively low (9.52 mg/100 g) as depicted in Table 3.

**TABLE 3:** Macronutrient, crude fibre and vitamin C contents of BobyGuard C.

Formulation	Proximate composition (g/100 g DM)					
	Protein	Carbohydrate	Lipid	Crude fibre	Energy (Kcal/100 g DM)	Vitamin C (mg/100 g DM)
BGC	38.36 ± 0.02	59.70 ± 0.62	3.16 ± 0.47	2.46 ± 0.10	420.70 ± 1.71	9.52 ± 0.20

Values are expressed as mean ± SEM.

### 3.3 Mineral content of BobyGuard C

The mineral composition of BobyGuard C is highlighted in Table 4, showing significant amounts of magnesium (60,066.67 µg/100 g), manganese, zinc, and copper, among others. However, heavy metals such as lead, mercury, cadmium, and arsenic were not detected, indicating the safety of the formulation concerning toxic metal contamination.

**TABLE 4:** Minerals and heavy metals contents of BobyGuard C.

Mineral (µg/100 g)	BGC
Magnesium	60,066.67 ± 1819.17
Manganese	13,886.67 ± 479.07
Zinc	15,323.33 ± 419.54
Copper	6,350.00 ± 0.00
Potassium	6,847.00 ± 5.03
Chlorine	403.33 ± 3.48
Iron	315.33 ± 2.03
Phosphorus	204.67 ± 1.67
Sulphur	236.00 ± 1.16
Lead	ND
Mercury	ND
Cadmium	ND
Arsenic	ND

ND (not detected); Values are expressed as mean ± SEM.

### 3.4 Phytochemical characteristic of BobyGuard C

Compounds such as phenols, flavonoids, alkaloids, tannins, glycosides, triterpenes, anthocyanins, and sugars were detected in BGC (Table 5). However, steroids and saponins were not detected in the formulation, suggesting that BobyGuard C is rich in bioactive compounds, which may contribute to its potential therapeutic effects.

**TABLE 5:** Qualitative phytochemical screening of entrants, BobyGuard C.

Test	Phenol	Flavonoid	Alkaloid	Tannins	Steroids	Saponin	Glycoside	Triterpene	Anthocyanin	Sugars
BGC	+	+	+	+	-	-	+	+	+	+

+: Detected, -: not detected.

### 3.5 Phytochemical content of BobyGuard C

Various phytochemicals were present in relatively high quantities in BobyGuard C, including phenols (9,783.48 mg GAE/100 g), flavonoids (47.72 mg eq QuE/100 g), alkaloids (106.14 mg eq berberine/100 g), and carotenoids (196.86 mg/100 g) (Table 6).

**TABLE 6:** Phytochemical content of BobyGuard C.

Formulation	Phenol (mg GAE/100 g DM)	Flavonoids (mg eq QuE/100 g DM)	Flavonols (mg eq QuE/100 g DM)	Alkaloids (mg eq berberine/100 g DM)	Tannin (mg eq leucocyandine/100 g DM)	Oxalate (mg/100 g DM)	Carotenoids (mg/100 g DM)	Phytate (mg eq phytic acid/100 g DM)
BGC	9,783.48 ± 316.22	47.72 ± 3.38	3.88 ± 0.12	106.14 ± 2.20	9.46 ± 0.08	11.29 ± 0.39	196.86 ± 0.45	1.45 ± 0.12

Values are expressed as mean ± SEM.

### 3.6 Bioactive compounds in BobyGuard C using LC–MS/MS

**FIGURE 1:**
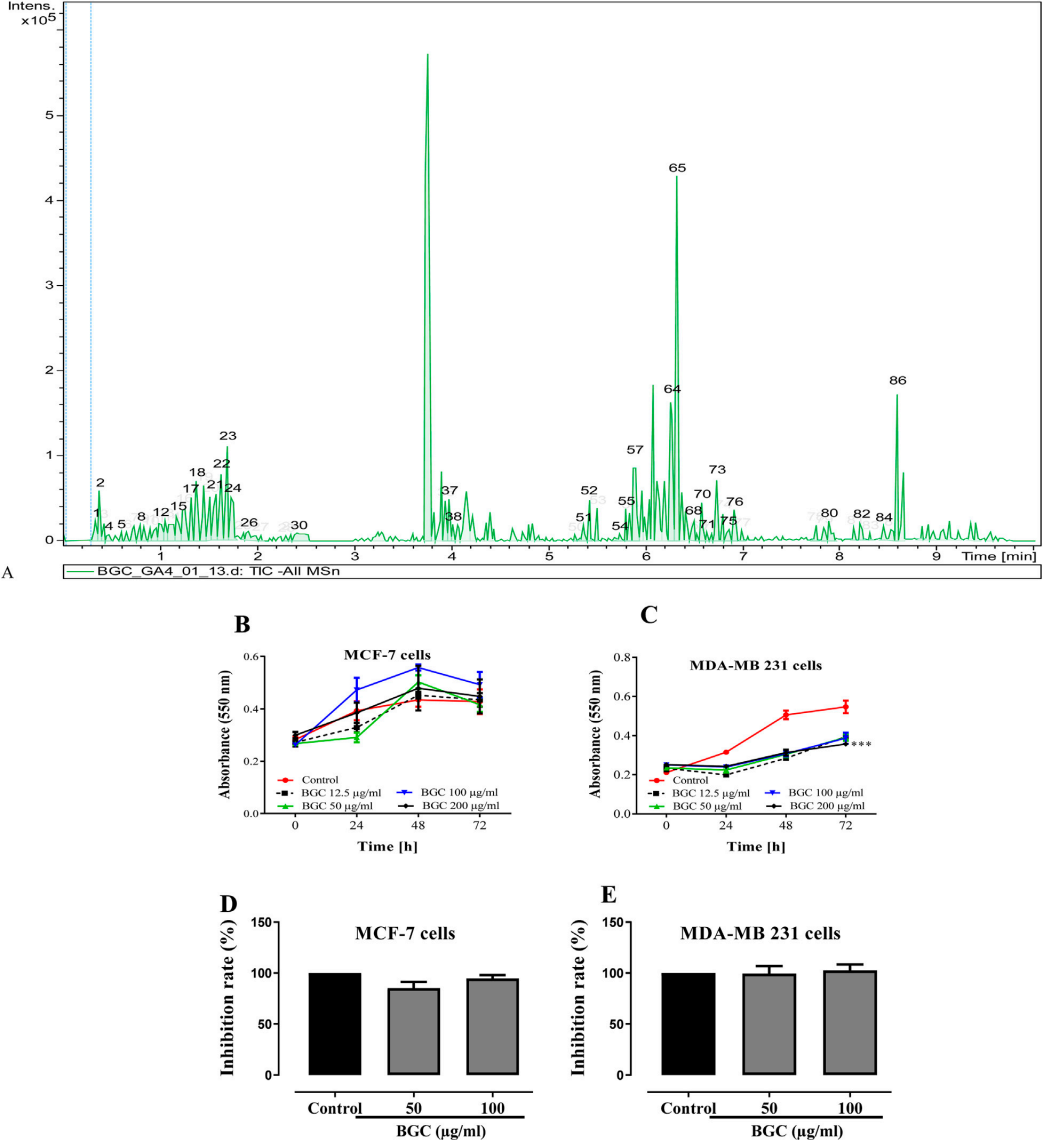
**(A)** Base peak chromatogram for methanol extract in (−)-ESI mode, portraying annotated compounds from BGC. Growth of estrogen sensitive MCF-7 **(B)** and non-sensitive MDA-MB 231 **(C)** breast carcinoma cells treated with different concentrations of BGC after 24, 48 and 72 h; Cell proliferation of estrogen sensitive MCF-7 **(D)** and non-sensitive MDA-MB 231 **(E)** breast carcinoma cells. Cells were treated with BGC at 5, 10 and 20 μg/mL after 48 h. Controls remained untreated (n = 3). Treated cancer cell cultures were compared to non-treated control cultures of the same passage and cell numbers per well. ***p < 0.001 compared to control.

Table 7 presents the different bioactive secondary metabolite compounds present in BGC, characterized by the presence of 21 identified compounds (Figure 1A) including Quercetin 7-xyloside., Afrostyraxthioside A, Diosbulbin H and Diosbulbin B.

**TABLE 7:** Secondary metabolites in BobyGuard C annotated in negative mode.

Peak	RT	M-H [m/z]	MS/MS fragment ions [m/z]	Formula	Suggested compound	Metabolite class	Biological activty	Height	RDBE
1	0.63	152.9973	78.9381 90.9963 96.9364 107.9999	C_7_H_7_NO_3_	2-Amino-3-hydroxybenzoic acid	Amino acid derivative	Antioxidant	21,548.94	5
2	0.96	579.1612	109.0043 125.0023 128.0126 137.0022 145.0244 152.9975	C_40_H_52_O_3_	Adonirubin	Carotenoid	Antioxidant, anti-inflammatory	3,271.75	15
3	1.14	281.0926	109.0079 123.0234 137.0020 146.0138 151.0195	C_17_H_14_O_4_	5-[1-(3,4-Dihydroxyphenyl)ethyl]cyclopenta[c]pyran-7-carboxaldehyde	Flavonoid derivative	Antioxidant	1,345.94	11
4	1.29	577.1311	109.0125 125.0026 137.0036 151.0233	C_30_H_26_O_12_	Proanthocyanidin B2	Flavonoid	Antioxidant, anti-cancer	11,045.44	15
5	1.42	289.0561	109.0063 117.0208 123.0225 128.0124 145.0085	C_15_H_14_O_6_	Catechin	Flavan-3-ol	Antioxidant, anti-inflammatory	62,393.00	8
6	202	413.1629	85.0066 89.0022 98.9847 101.0023 113.0032 130.9447	C_27_H_42_O_3_	diosgenin	Steroidal saponin	Anti-inflammatory, anticancer	3,858.56	7
7	3.22	301.1219	84.0230 93.0140 96.9336 109.0092 130.0635 147.0257	C_15_H_10_O_7_	quercetin	Flavonoid	Antioxidant, anti-inflammatory	3,299.31	10
8	3.16	387.1027	93.0174 96.9454 101.0041 112.9630 119.0163 130.0648 137.0033	C_20_H_20_O_8_	Bafoudiosbulbin B	Glycoside	Cytotoxic	1741.94	10
9	3.92	433.0623	96.9393 112.9642 123.0248 130.0642 140.0264 150.9823 175.0216	C_20_H_18_O_11_	Quercetin 7-xyloside	Flavonoid glycoside	Antioxidant	12,160.69	10
10	3.96	447.0811	152.9996 157.0419 165.0331 174.0467 179.0590	C_21_H_20_O_11_	Kaempferol-3-O-hexoside	Flavonoid glycoside	Antioxidant, anticancer	6,819.38	11
11	4.14	255.0177	68.9745 79.9373 89.9025 93.0132 96.9401 112.9641	C_8_H_16_O_5_S_2_	Afrostyraxthioside A	Thioside	Antioxidant	2,160.25	0
12	4.26	417.1079	89.0016 93.0064 109.0103 112.9646 131.0310	C_23_H_30_O_7_	Diosbulbin H	Terpenoid	Antitumor	3,513.50	8
13	5.51	343.0623	112.9649 123.9079 134.0168 139.0876 149.0414 158.0149	C_19_H_20_O_6_	Diosbulbin B	Terpenoid	Antitumor	1,343.75	9
14	5.97	569.2661	112.9630 144.5204 152.9759 240.9932 279.2140 315.0388	C_33_H_30_MgN_4_O_4_	Pyrochlorophyllide b	Chlorophyll derivative	Antioxidant	6,222.56	15
15	6.17	595.2823	272.5279 279.2195 293.2040 311.1561 315.0254	C_40_H_52_O_4_	Astaxanthin	Carotenoid	Antioxidant, anti-inflammatory		13
16	6.35	535.3067	80.9438 164.9691 224.9905 253.1999 299.0286	C_40_H_56_	β-carotene	Carotenoid	Antioxidant, vision health	3,431.56	14
17	6.29	555.2752	80.9431 164.9676 224.9885 255.2193 299.0249	C_33_H_32_MgN_4_O_3_	Pyrochlorophyllide a	Chlorophyll derivative	Antioxidant	31,165.88	16
18	6.36	295.2132	96.9399 112.9637 182.9964 277.2104	C_17_H_12_O_5_	Diosbulbiol A	Terpenoid	Antioxidant, anticancer	2,472.31	11
19	6.42	561.3266	78.9379 96.9464 253.0761 279.2181 315.0413	C_40_H_50_O_2_	Rhodoxanthin 1	Carotenoid	Antioxidant	5,246.06	16
20	6.75	537.3196	255.2160	C_26_H_34_O_12_	Diosbulbinoside F	Glycoside	Antioxidant	18,279.69	8
21	7	461.2550	78.9408 112.9632 152.9756 199.0183 255.2142 279.2154 2,925.2160 325.2153	C_23_H_26_O_10_	Bafoudiosbulbin G	Glycoside	Cytotoxic	1,557.31	10

### 3.7 Antioxidant and antiradical activities of BobyGuard C

Table 8 reports the antioxidant capacity of BobyGuard C using different assays, including DPPH (90.39%), FRAP (1,450.18 mg eq FeSO_4_/100 g), OH scavenging activity (34.85%), and total antioxidant capacity (20.02 mg eq ascorbic acid/100 g). These values indicate that BobyGuard C has strong antioxidant properties.

**TABLE 8:** Antioxidant and antiradical activities of BobyGuard C.

Formulation	DPPH (%)	FRAP (mg eq FeSO_4_/100 g DM)	OH (%)	TAC (mg eq ascorbic acid/100 g DM)
BGC	90.39 ± 0.33	1,450.18 ± 15.10	34.85 ± 0.83	20.02 ± 0.24

Values are expressed as mean ± SEM.

### 3.8 Inhibitory effect of BobyGuard C on cell growth and proliferation

The MTT assay is a widely used colorimetric assay for assessing cell metabolic activity, which serves as an indirect measure of cell growth and proliferation. In this study, we employed the MTT assay to compare the proliferation rates of estrogen-sensitive (ER-positive) and estrogen-insensitive (ER-negative) breast cancer cells. The anti-growth (Figures 1B,C) and anti-proliferative (Figures 1D,E) effects of BGC on MCF-7 and MDA-MB 231 cell lines were determined. The effect of BGC on cell growth revealed a dose-independent and time-independent decreases in both non-invasive MCF-7 (hormone-dependent) and invasive MDA-MB 231 (hormone-independent) breast carcinoma cell lines. However, BGC showed significantly greater (p < 0.001) anti-growth potential on MDA-MB 231 cell lines at all tested concentrations. While it had a non-significant and antiproliferative activity on both cell lines, even though it was more potent on MCF-7 cells.

### 3.9 Acute toxicity effect of BobyGuard C on survival, weight, behavioral signs and organs weight

After administration of a single dose of 5,000 mg/kg of BGC, the animals were observed for 14 days. The study monitored various behaviors, such as fur condition, noise sensitivity, mobility, grooming, fecal state, and aggressiveness. No behavioral signs of toxicity and no mortality were observed in any group during the experiment (Table 9).

**TABLE 9:** Behavioral signs of rats treated with a single dose of BGC (5,000 mg/kg).

Time	30 min		2H		4H		24H		48H		Week 1		Week 2	
Observation	C	D	C	D	C	D	C	D	C	D	C	D	C	D
Fur	N	N	N	N	N	N	N	N	N	N	N	N	N	N
Sensitivity to noise	N	N	N	N	N	N	N	N	N	N	N	N	N	N
Mobility	N	N	N	N	N	N	N	N	N	N	N	N	N	N
Grooming	N	N	N	N	N	N	N	N	N	N	N	N	N	N
State of faeces	N	N	N	N	N	N	N	N	N	N	N	N	N	N
Aggressivity	N	N	N	N	N	N	N	N	N	N	N	N	N	N
Dead	A	A	A	A	A	A	A	A	A	A	A	A	A	A
Tumor	A	A	A	A	A	A	A	A	A	A	A	A	A	A

NOR (receiving 2% ethanol (vehicle)); BGC5000 (receiving BobyGuard C orally at a dose of 5,000 mg/kg daily).

C = normal control; D = 5,000 mg/kg; N = normal; A = absent.

**FIGURE 2:**
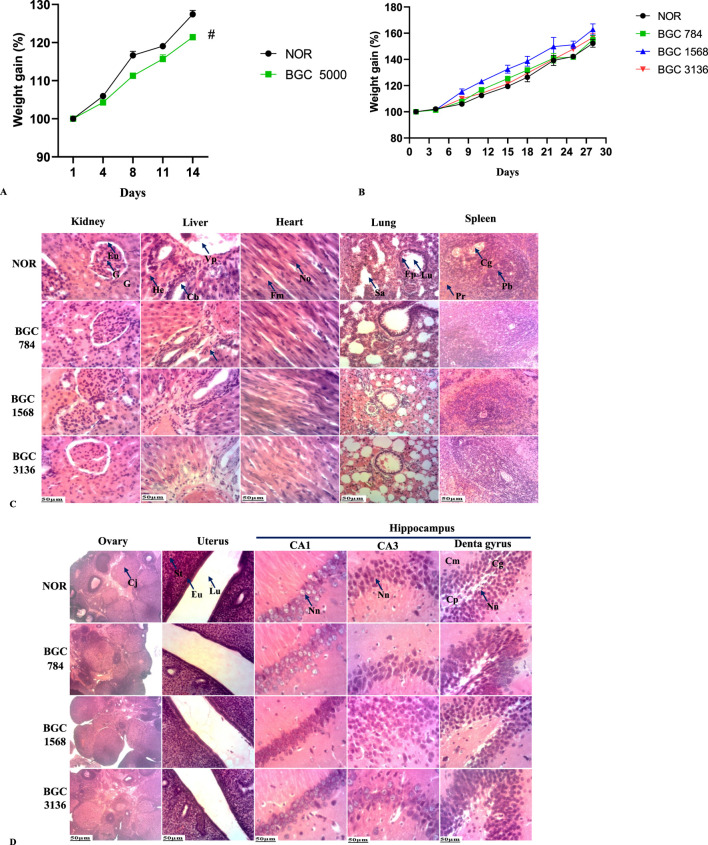
**(A)** Bodyweight of rats treated with a single dose of BobyGuard C (5000 mg/kg). **(B)** Weight change of BobyGuard **(C)** treated rats in sub-acute toxicity study. **(C)** Kidney, liver, heart, lung and spleen microarchitectures after treatment with BobyGuard C (784, 1,568 and 3,136 mg/kg). **(D)** Ovary, uterus and hippocampus microarchitectures after treatment with BobyGuard C (784, 1,568 and 3,136 mg/kg) BGC5000 (receiving BobyGuard C orally at a dose of 5,000 mg/kg daily). NOR (receiving 2% ethanol (vehicle)); BGC784 (receiving BobyGuard C orally at a dose of 784 mg/kg daily); BGC1568 (receiving BobyGuard C orally at a dose of 1,568 mg/kg daily) and BGC3136 (receiving BobyGuard C orally at a dose of 3,136 mg/kg daily). #*p <* 0.05, ##*p <* 0.01 and ###*p <* 0.001 compared to the normal control (NOR). Kidney: G = Glomerulus, Eu = Urinary Space, Liver: Vp = Portal Vein, He = Hepatocyte, Cb = Bile Duct, Ah = Hepatic Artery. Spleen: Pb = White Pulp, Pr = Red Pulp, Lung: Ep = Pulmonary Epithelium, Sa = Alveolar Sac. CA1: Nn = Normal Neuron; CA3: Nn = Normal Neuron, Dentate Gyrus: Cg = Granule Cell Layer, Cp = Polymorphic Cell Layer, Cm = Molecular Layer. Uterus: Lu = Uterine Lumen; En = Endometrium; St = Stroma.

As shown in Figure 2A, body weight continually increased over time in both groups, although it was more prominent in the normal control group (*p <* 0.05).

Also, at necropsy, significant macroscopic changes in the size and weight of the spleen (#p < 0.05), uterus (#p < 0.05), lungs (##p < 0.01) and stomach (###p < 0.001) were observed (Table 10). No signs of distress or death were registered, indicating that the LD_50_ is greater than 5,000 mg/kg.

**TABLE 10:** Organs weight of rats treated with a single dose of BGC (5,000 mg/kg).

Organs (g/100 g)	NOR	BGC5000
Heart	0.38 ± 0.01	0.35 ± 0.02
Lungs	0.63 ± 0.02	0.86 ± 0.06^##^
Liver	3.40 ± 0.13	3.28 ± 0.11
Kidney	0.59 ± 0.01	0.60 ± 0.04
Spleen	0.43 ± 0.02	0.48 ± 0.01^#^
Adrenals	0.03 ± 0.00	0.03 ± 0.00
Stomach	0.80 ± 0.04	0.96 ± 0.07^###^
Brain	0.94 ± 0.02	1.01 ± 0.06
Ovaries	0.06 ± 0.01	0.06 ± 0.00
Uterus	0.22 ± 0.02	0.15 ± 0.01^#^

Values are expressed as mean ± SEM. NOR (receiving 2% ethanol (vehicle)); BGC5000 (receiving BobyGuard C orally at a dose of 5,000 mg/kg daily). #p < 0.05, ##p < 0.01 and ###p < 0.001 compared to the normal control (NOR).

### 3.10 Subacute toxicity effect of BobyGuard C on survival, weight and hematological parameters

No deaths or significant differences in body weight were observed in any of the treated groups compared to the normal group (Figure 2B).

Table 11 shows the effects of different doses of BobyGuard C (784 mg/kg, 1,568 mg/kg, and 3,136 mg/kg) on the blood parameters of rats. All BGC groups showed a significant increase (*p <* 0.01), with BGC 1568 peaking at 11.23 ± 0.38, indicating a possible inflammatory or immune response. RBC counts also increased, with BGC 1568 reaching 8.14 ± 0.12 (10^6/µL) before dropping in BGC 3136. Hemoglobin levels rose across all BGC groups, highest in BGC 1568 at 14.67 ± 0.13 g/dL, aligning with the RBC data and suggesting enhanced oxygen transport capacity. Platelet counts remained stable. MCH and MCHC values were consistent across groups, indicating stable hemoglobin content and concentration in RBCs. Lymphocyte percentages decreased significantly in BGC groups, especially in BGC 1568 and BGC 3136, indicating shifts in leukocyte dynamics. Monocyte percentages rose markedly, with BGC 1568 suggesting possible chronic inflammation or immune activation. At all treated doses, slight to moderate toxicity severity was observed for all the evaluated hematological parameters, except for WBC which showed severe deviation at doses of 1,568 mg/kg and 3,136 mg/kg, indicating immune system stimulation or bone marrow damage or activation.

**TABLE 11:** Hematological parameters of BGC- treated rats in sub-acute toxicity study.

Parameter	NOR	BGC 784	BGC 1568	BGC 3136
WBC (10^3^/µL)	5.97 ± 0.19	7.90 ± 0.07^##^	11.23 ± 0.38^###^	11.00 ± 0.38^###^
RBC (10^6^/µL)	6.93 ± 0.48	7.73 ± 0.10	8.14 ± 0.12^#^	7.53 ± 0.31
Hemoglobin (g/dL)	12.50 ± 0.91	14.00 ± 0.21	14.67 ± 0.13	13.33 ± 0.63
Platelet (10^3^/µL)	545.00 ± 9.02	536.67 ± 9.24	587.00 ± 11.14^#^	524.67 ± 19.68
MCH (pg)	18.00 ± 0.06	18.13 ± 0.38	18.03 ± 0.20	17.77 ± 0.54
MCHC (g/dL)	30.27 ± 0.43	30.90 ± 0.32	30.97 ± 0.37	30.47 ± 0.30
MCV (fL)	59.57 ± 1.08	58.60 ± 1.49	58.20 ± 0.17	58.33 ± 1.34
HCT (%)	41.39 ± 1.61	45.28 ± 0.14^#^	47.37 ± 0.67^##^	43.90 ± 0.29
PCT (%)	0.41 ± 0.01	0.40 ± 0.04	0.43 ± 0.01	0.38 ± 0.04
Lymphocyte (%)	80.20 ± 0.60	78.73 ± 1.66	73.43 ± 0.39^##^	72.67 ± 0.09^###^
Monocyte (%)	8.97 ± 0.30	9.37 ± 0.24	15.93 ± 0.03^###^	14.00 ± 0.30^###^
Granulocyte (%)	10.83 ± 0.12	11.90 ± 0.30	10.63 ± 0.22	13.33 ± 0.42^###^
Deviations from normal values (%)				
WBC	—	32.33	88.11	84.25
RBC	—	11.54	17.46	8.66
Hemoglobin	—	12.00	17.36	6.64
Platelet	—	−1.53	7.71	−3.73
MCH	—	0.72	0.17	−1.28
MCHC	—	2.08	2.31	0.66
MCV	—	−1.63	−2.30	−2.08
HCT	—	9.40	14.45	6.06
PCT	—	−2.44	4.88	−7.32
Lymphocyte	—	−1.83	−8.44	−9.39
Monocyte	—	4.46	77.59	56.08
Granulocyte	—	9.88	−10.67	25.40

Values are expressed as mean ± SEM. NOR (receiving 2% ethanol (vehicle)); BGC784 (receiving BobyGuard C orally at a dose of 784 mg/kg daily); BGC1568 (receiving BobyGuard C orally at a dose of 1,568 mg/kg daily) and BGC3136 (receiving BobyGuard C orally at a dose of 3,136 mg/kg daily). #p < 0.05, ##p < 0.01 and ###p < 0.001 compared to the normal control (NOR). (−) = decrease, (+) = increase.

### 3.11 Subacute toxicity effect of BobyGuard C on some biochemical parameters

Table 12 examines the impact of BobyGuard C on various biochemical markers, including protein, albumin, globulin, ALT, AST, ALP, creatinine, triglycerides, and cholesterol. Significant changes were observed in ALT, triglycerides, and cholesterol levels at different doses, indicating possible effects on liver function and lipid metabolism. Total protein levels remained relatively stable, with BGC 3136 showing a slight increase, while albumin levels varied in a dose-dependent manner. The albumin/globulin ratio varied significantly, indicating changes in liver function or protein metabolism. ALT levels increased significantly in BGC 784, while AST and ALP levels were elevated in BGC 1568 and BGC 3136. Triglyceride levels decreased significantly in all BGC groups, suggesting improved lipid metabolism, while at all doses, it significantly (*p <* 0.001) increased total cholesterol levels.

**TABLE 12:** Biochemical parameters of BGC- treated rats in sub-acute toxicity study.

Parameter	NOR	BGC 784	BGC 1568	BGC 3,136
Protein (g/dL)	3.87 ± 0.02	3.86 ± 0.08	3.81 ± 0.07	4.02 ± 0.03
Albumin (g/dL)	1.59 ± 0.02	1.45 ± 0.02	1.55 ± 0.04	1.72 ± 0.07
Globulin (g/dL)	2.27 ± 0.05	2.41 ± 0.02	2.27 ± 0.04	2.29 ± 0.07
Albumin/Globulin	0.70 ± 0.02	0.62 ± 0.02^#^	0.70 ± 0.03	0.79 ± 0.02
ALT (U/L)	32.43 ± 0.84	57.63 ± 3.00^###^	36.63 ± 0.43^##^	36.17 ± 0.76^##^
AST (U/L)	160.65 ± 3.88	149.52 ± 3.42^###^	149.73 ± 1.43^###^	161.24 ± 3.52
ALP (U/L)	231.44 ± 2.61	203.72 ± 3.49^###^	179.96 ± 2.51^###^	231.55 ± 8.02
Creatinine (mg/dL)	1.67 ± 0.08	1.42 ± 0.02^###^	1.56 ± 0.05^#^	1.63 ± 0.03
Triglyceride (mg/dL)	242.78 ± 2.34	152.64 ± 2.66^###^	158.25 ± 2.89^###^	177.75 ± 3.30^###^
Total cholesterol (mg/dL)	23.72 ± 0.27	22.20 ± 0.45	27.49 ± 0.78^###^	42.86 ± 4.05^###^

Values are expressed as mean ± SEM. NOR (receiving 2% ethanol (vehicle)); BGC784 (receiving BobyGuard C orally at a dose of 784 mg/kg daily); BGC1568 (receiving BobyGuard C orally at a dose of 1,568 mg/kg daily) and BGC3136 (receiving BobyGuard C orally at a dose of 3,136 mg/kg daily). #p < 0.05, ##p < 0.01 and ###p < 0.001 compared to the normal control (NOR).

### 3.12 Subacute toxicity effect of BobyGuard C on organs weight and microscopic architecture

Table 13 reveals that BGC treatment resulted in significant, dose-dependent changes in the weights of various organs. The heart, lungs, liver, spleen, and ovaries exhibited significant weight changes with increasing doses of BGC. Specifically, heart and uterine weights decreased, while lung, liver, spleen, and ovarian weights increased significantly (*p <* 0.001). The adrenal glands and stomach showed selective weight changes at certain doses, suggesting some variability in sensitivity.

**TABLE 13:** Organs weight of BGC- treated rats in sub-acute toxicity study.

Organ (g/100 g)	NOR	BGC 784	BGC 1568	BGC 3,136
Heart	0.38 ± 0.01	0.33 ± 0.00^###^	0.34 ± 0.00^###^	0.35 ± 0.00^##^
Lung	0.70 ± 0.01	0.85 ± 0.00^###^	0.78 ± 0.01^###^	0.88 ± 0.01^###^
Liver	3.77 ± 0.11	3.93 ± 0.08	4.13 ± 0.04^##^	4.63 ± 0.02^###^
Kidney	0.69 ± 0.06	0.68 ± 0.00	0.66 ± 0.00	0.64 ± 0.00
Spleen	0.45 ± 0.02	0.67 ± 0.00^###^	0.63 ± 0.00^###^	0.68 ± 0.00^###^
Adrenals	0.03 ± 0.00	0.04 ± 0.00^###^	0.03 ± 0.00	0.03 ± 0.00^##^
Stomach	0.89 ± 0.01	0.88 ± 0.00	0.98 ± 0.01^###^	0.91 ± 0.00
Brain	1.04 ± 0.03	1.03 ± 0.01	0.84 ± 0.01^###^	0.96 ± 0.03
Ovaries	0.06 ± 0.00	0.06 ± 0.00^#^	0.07 ± 0.00^#^	0.07 ± 0.00^###^
Uterus	0.23 ± 0.01	0.17 ± 0.00^###^	0.26 ± 0.00^###^	0.15 ± 0.00^###^

Values are expressed as mean ± SEM. NOR (receiving 2% ethanol (vehicle)); BGC784 (receiving BobyGuard C orally at a dose of 784 mg/kg daily); BGC1568 (receiving BobyGuard C orally at a dose of 1,568 mg/kg daily) and BGC3136 (receiving BobyGuard C orally at a dose of 3,136 mg/kg daily). #p < 0.05, ##p < 0.01 and ###p < 0.001 compared to the normal control (NOR).

The microscopic appearance of the lung, liver, and kidney in both control and treated groups showed smooth surfaces and soft consistency. Light microscopy images after H&E staining confirmed that no morphological or histological lesions were present in the organs of BGC-treated rats at any dose level as shown in Figures 2C,D.

## 4 Discussion

Optimizing formulations enhances the bioavailability and stability of active ingredients, ensuring their effectiveness throughout the product’s shelf life. Achieving a potent and reliable polyherbal formulation requires a careful balance of components. Extraction yield plays a key role in obtaining bioactive compounds like alkaloids, glycosides, phenolics, saponins, and tannins, which contribute to antioxidant, anti-inflammatory, and antibacterial properties (Sultana et al., 2009). Standardizing herbal medicines with defined qualitative and quantitative measures ensures quality, safety, and efficacy. Chemical tests confirmed the presence of glycosides, tannins, flavonoids, alkaloids, and other phytochemicals, which help combat oxidative stress-related conditions such as inflammation, cardiovascular diseases, and cancer. These compounds also influence enzyme activity, including paraoxonases, impacting inflammation and oxidative stress pathways (Arab et al., 2022). Flavonoids, alkaloids, and saponins exhibit antioxidative properties, regulating cell functions and offering therapeutic potential against cancer and diabetes (Lim and Park, 2022).

Preliminary physicochemical analysis provided insights into the polyherbal nutraceutical. The pH remained acidic, which may influence microbial contamination and drug absorption (Ellison, 2002). Moisture content was within the acceptable range (8%–14%), which is crucial for stability and preventing microbial growth (Kaur and Sharma, 2022). Ash value, a marker of purity, suggested no significant contamination (Knevel et al., 1982; Chandel et al., 2011). The formulation showed solubility in water, ethanol, and ethyl acetate, with higher extraction efficiency in water than alcohol. Such variations impact extractive values, ultimately influencing the formulation’s activity (Jahan et al., 2008).

BGC contains proteins, carbohydrates, and vitamin C, making it a valuable nutritional component. A well-balanced diet that replenishes energy, protein, and essential nutrients is more effective than relying on a single nutrient (Arends et al., 2016). Spirulina, a key ingredient, is rich in proteins, essential amino acids, vitamins, and polyunsaturated fatty acids, contributing to its high nutritional value (Bortolini et al., 2022; Ngo Matip et al., 2022). Higher intake of vitamin C, carotenoids, and α-tocopherol is linked to reduced risks of cardiovascular disease, cancer, and overall mortality (Aune et al., 2018). Protein intake is particularly important for individuals with chronic diseases (Markovic and Natoli, 2009). Plant-based proteins, along with bioactive compounds like tannins, flavonoids, and fibers, help combat these conditions (Cavazos and De Mejia, 2013; Ramamurthy et al., 2012). Dietary fibers further support health by improving lipid profiles (Surampudi et al., 2016) and enhancing immune function (Watzl et al., 2005). Vitamin C plays essential roles in collagen synthesis, immune function, iron absorption, and antioxidant defense (Chen et al., 2008). Patients on high-protein, low-fat diets often face deficiencies in key minerals like iron, calcium, magnesium, zinc, and copper (Astrup and Bügel, 2018). BGC contains essential macro- and microminerals, including magnesium, phosphorus, potassium, iron, and zinc, while heavy metals such as mercury, cadmium, arsenic, and lead were absent. These minerals play vital roles in antioxidant activity, inflammation control, and enzymatic functions. For example, zinc supports over 300 enzymes, aiding in antioxidant defense and cell regulation (Nimmanon and Taylor, 2019). Potassium and sodium balance is crucial for cellular function, while magnesium regulates energy metabolism, blood pressure, and neural signaling (Michalczyk and Cymbaluk-Płoska, 2020). Iron is essential for oxygen transport and immune function, while copper supports cardiovascular health and oxidative defense (Wu et al., 2022; Liu et al., 2023). Inorganic sulfur also helps protect cells from DNA damage (Zhang et al., 2014). Importantly, all minerals in BGC remained within safe limits set by the WHO (2007), ensuring its nutritional safety.

Secondary metabolites, particularly flavonoids and phenolic compounds, are well known for their antioxidant and antiradical activities. Their hydroxyl groups and conjugated ring structures allow them to stabilize free radicals (Seyoum et al., 2006). The high levels of phenols, alkaloids, carotenoids, and flavonoids in both formulations significantly contribute to their antioxidant potential. Carotenoids exert their effects through hydrogen atom transfer (HAT), single-electron transfer (SET), and metal chelation, playing a key role in redox defense. Their antioxidant properties may help reduce cancer cell growth and induce apoptosis (Wei et al., 2023). Alkaloids also exhibit strong antioxidant activity, primarily through free radical quenching and electron transfer mechanisms. Their ability to stabilize free radicals enhances their potential in oxidative stress-related conditions. Additionally, alkaloids activate antioxidant pathways such as Nrf2/HO-1 (Neganova et al., 2012). Many compounds identified in BGC are associated with anti-inflammatory, anticancer, and analgesic effects. Alkaloids, in particular, are valuable for drug development, having demonstrated antiproliferative, anti-inflammatory, and antimetastatic properties against various cancers (Kurek, 2019). The medicinal efficacy of plant extracts is closely linked to their antioxidant properties. Since different antioxidant assays yield varying results due to differences in mechanisms and evaluation parameters, Schlesier et al. (2002) recommend using at least two methods. This study employed multiple *in vitro* assays (DPPH, TAC, FRAP, and OH) to comprehensively assess BGC’s antioxidant potential. BGC demonstrated strong antioxidant and antiradical activity across all assays. The DPPH assay confirmed its free radical scavenging capacity, highlighting the presence of phenolic compounds with electron transfer or hydrogen-donating abilities (Jing et al., 2012). The FRAP assay indicated significant ferric-reducing properties, further supporting its hydrogen-donating potential (Atere et al., 2018). The hydroxyl radical scavenging assay showed BGC’s ability to neutralize hydroxyl radicals, the primary contributors to oxidative damage (Jomova et al., 2023). Additionally, BGC exhibited a high total antioxidant capacity (TAC), aligning with evidence linking dietary TAC to reduced chronic disease risks. Studies have shown an inverse relationship between dietary TAC and aggressive prostate cancer, as well as lower breast cancer risk in postmenopausal women. Higher TAC intake has also been associated with improved disease-free survival after breast cancer surgery (Pellegrini et al., 2018). The scavenging activity of BGC was compared to that of ascorbic acid. At same concentration (100 μL/mL), ascorbic acid exhibited a 96.96% inhibition for DPPH method and 72.46% for OH method. BGC exhibited antioxidant activity comparable to that of a polyherbal extract from Vetiveria zizanioides, *Trichosanthes cucumerina* and Mollugo cerviana, a polyherbal formulation known to possess cytotoxic and antibacterial properties (Seshadri et al., 2020).

BGC was tested *in vitro* on estrogen-sensitive (ER-positive) and estrogen-insensitive (ER-negative) breast cancer cells using the MTT assay. Both cell lines exhibited a dose-independent decrease or no change in growth and proliferation when treated with BGC. However BGC demonstrated better antigrowth activity on estrogen-insensitive (ER-negative) breast cancer cells. These findings align with previous research suggesting that certain bioactive compounds preferentially inhibit the growth of more aggressive cancer cell lines through mechanisms such as the induction of apoptosis, cell cycle arrest, or inhibition of specific signaling pathways (Rahman et al., 2022). BGC’s stronger effect on inhibiting growth rather than directly inducing cell death indicates that it may primarily work by arresting cell cycle progression. While this may limit its effectiveness as a standalone cytotoxic agent, it opens up important opportunities for its use in growth-suppressive therapies, chemoprevention, or as an adjunct to enhance the efficacy of other treatments. Also, the efficacy of BGC, particularly against hormone-independent cancer cells, suggests that it could be a valuable component of combination therapies aimed at targeting multiple pathways and reducing the likelihood of resistance.

In the acute toxicity study, a single dose of 5,000 mg/kg of BGC did not cause mortality or severe toxicity in rats. This dose corresponds to approximately 810 mg/kg in humans, calculated using the formula (Reagan-Shaw et al., 2007):

$$\text{Human dose}=\text{Animal dose}\times \left(\text{Animal Km}/\text{Human Km}\right)$$

where Km for a 0.15 kg rat is 6, and for a 60 kg human, it is 37. Therefore, the estimated median lethal dose (LD_50_) of BGC is higher than 810 mg/kg for humans, which is equivalent to about 48.6 g for a 60 kg adult. According to toxicity classification, substances with LD_50_ values of 1–5 g/kg are considered to have low toxicity, while those exceeding 5 g/kg are generally classified as non-toxic (Piyachaturawat et al., 2002).

In the sub-acute toxicity test, BGC was administered for 28 days without affecting survival, body weight, or the histology of vital organs (liver, kidney, heart, brain). However, serum biochemical parameters showed significant alterations in kidney function (creatinine), liver function (AST, ALT, ALP, albumin), and lipid profile (total cholesterol), though these changes were not dose-dependent. Notably, even the highest dose (3,136 mg/kg/day) did not consistently affect biochemical markers, except for cholesterol. This aligns with the low-dose hypothesis, which suggests that biological responses at minimal exposure can differ qualitatively from those at higher doses (Borgert et al., 2021). Conversely, high doses may not always cause toxicity due to enhanced metabolism and elimination (Sewell et al., 2022). Despite stable biochemical markers, organ weights (lungs, liver, spleen) significantly increased at higher doses, suggesting compensatory changes such as hypertrophy or hyperplasia, which may occur without immediate biochemical alterations (Sellers et al., 2007). Since blood components are frequently exposed to circulating substances, hematological parameters provide critical toxicity insights. In this study, RBC and WBC levels significantly increased in a dose-related manner, likely due to the hemopoietic and immune-stimulatory effects of BGC. A “left shift” pattern was observed, with increased granulocytes and monocytes but reduced lymphocytes. This shift suggests BGC may enhance innate immunity by elevating neutrophils and monocytes, which are crucial for inflammation and pathogen defense. The lymphocyte reduction may indicate an altered immune balance, possibly due to adaptive immune modulation or redistribution to peripheral tissues. The excessive dose dependent leukocytosis in response to BGC may indicate underlying chronic inflammation or immune dysregulation. Such an immune overactivation carries the risk of long-term complications, including tissue damage, the development of autoimmune conditions, and progressive organ dysfunction. The organs most vulnerable to excessive or sustained leukocytosis include the bone marrow, liver, spleen, kidneys, lungs, gastrointestinal tract, skin, and potentially the heart. Notably, the accompanying increase in ALT levels observed suggests that BGC may be inducing hepatic stress, further supported by hepatomegaly. These effects are likely driven by BGC’s bioactive compounds, such as flavonoids, alkaloids, and terpenoids, which are known for their immunomodulatory properties (Maheshwari et al., 2022). Overall, repeated administration of BGC at 3,136 mg/kg (approximately 523 mg/kg in humans) for 28 days, eventhough, caused changes in some biochemical parameters and some organ hypertrophy, it did not induce histomorphological lessions.

## 5 Conclusion

BobyGuard C exhibits significant potential as a polyherbal nutraceutical for breast cancer management, combining a rich nutritional profile with potent antioxidant and anticancer properties. The formulation demonstrated cytotoxic effects against aggressive breast cancer cell lines while maintaining an overall safe toxicological profile in animal studies. Acute toxicity tests confirmed an LD_50_ greater than 5,000 mg/kg, suggesting minimal risk at conventional doses. Although some hematological and biochemical alterations were observed in the sub-acute study, no severe toxicological signs or histopathological abnormalities were detected. These results underscore the potential of BGC as a complementary dietary intervention for cancer prevention and management at adequate doses (784 mg/kg). However, further in-depth pre-clinical studies are essential to fully establish its long-term efficacy (90 days chronic toxicity study), safety, and therapeutic mechanisms at lower doses.

## Data Availability

The raw data supporting the conclusions of this article will be made available by the authors, without undue reservation.

## Abbreviations

ALP, Alkaline Phosphatase; ALT, Alanine Aminotransferase; ANOVA, Analysis of Variance; AOAC, Association of Official Analytical Chemists; AST, Aspartate Aminotransferase; BGC, BobyGuard C; BW, Body Weight; CNH, Cameroon National Herbarium; DPPH, 1,1-diphenyl-2-picrylhydrazyl; FRAP, Ferric Reducing Antioxidant Power; Hb, Hemoglobin; HCT, Hematocrit; LC-MS, Liquid chromatography-mass spectrometry; MCH, Mean Corpuscular Hemoglobin; MCHC, Mean Corpuscular Hemoglobin Concentration; MCV, Mean Corpuscular Volume; OECD, Organisation for Economic Co-operation and Development; PLT, Platelet; RBC, Red Blood Cell; SEM, Standard Error of the Mean; TAC, Total Antioxidant Capacity; UHPLC, Ultra High-Performance Liquid Chromatography; WBC, White Blood Cell.
